# Formulation and Evaluation of Celastrol-Loaded Liposomes

**DOI:** 10.3390/molecules16097880

**Published:** 2011-09-13

**Authors:** Jie Song, Feng Shi, Zhenhai Zhang, Fenxia Zhu, Jing Xue, Xiaobin Tan, Luyong Zhang, Xiaobin Jia

**Affiliations:** 1 Key Laboratory of Delivery Systems of Chinese Meteria Medica, Jiangsu Provincial Academy of Chinese Medicine, Nanjing 210028, Jiangsu, China; Email: momo198420@163.com (J.S.); davidpharm@yeah.net (Z.Z.); zfxcjq@126.com (Z.F.); szxuejing@hotmail.com (J.X.); njtxb@hotmail.com(X.T.); 2 Department of Traditional Chinese Medicine, Shanghai University of Traditional Chinese Medicine, Shanghai 201203, China; Email: shifeng_1985_wcl@163.com (F.S.); 3 National Center of Drug Screening, China Pharmaceutical University, Nanjing 210038, Jiangsu, China; Email: lyzhang@cpu.edu.cn (L.Z.)

**Keywords:** celastrol, liposomes, rat intestine perfusion model, ethanol-injection method

## Abstract

The main purpose of this study was to evaluate the intestinal absorption and the antineoplastic effect of the poorly water-soluble drug celastrol when liposomes were used as oral drug delivery system. Liposomes were prepared by the ethanol-injection method. An optimized liposome formulation composed of phospholipid, cholesterol and Tween-80 resulted in favorable encapsulation efficiency at 98.06 ± 0.94%. Homogeneous and stable particle size of 89.6 ± 7.3 nm and zeta potential of −(87.7 ± 5.8) mV were determined by laser particle size analyzer. Subsequently, the four-site perfusion rat intestinal model revealed that celastrol-loaded liposomes had improved effective permeability compared to the free drug in four intestinal segments (p < 0.05). Moreover, celastrol-loaded liposomes could also inhibit the tumor growth in C57BL/6 mice. These results suggest that liposomes could be a promising perioral carrier for celastrol.

## 1. Introduction

**Figure 1:**
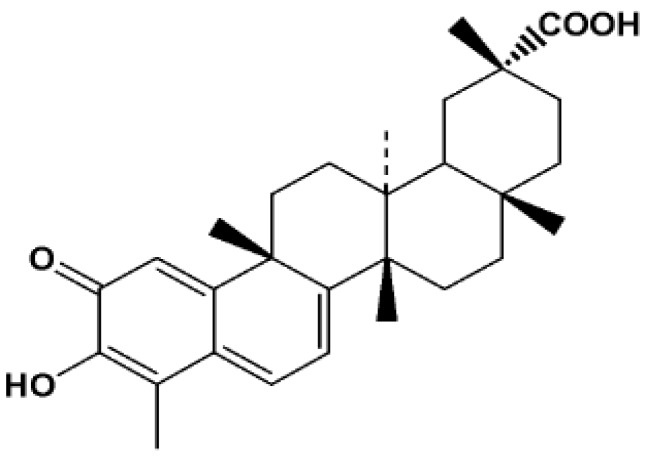
Chemical structure of celastrol.

Celastrol (Figure 1, also known as tripterine) is a major biologically active component extracted from the traditional Chinese medicinal herb, Tripterygium wilfordii Hook (also known as Thunder of God Vine). In recent years, it has attracted interest for its potential antitumor effects. Various cancer cell lines including C6 glioma cells, RPMI 8266 myeloma cells, K-562, pancreatic cancer cells, human chronic myelogenous leukemia, etc. are reported to be inhibited by celastrol [1,2,3,4,5]. However, its low aqueous solubility impedes the clinical use of celastrol. Only intraperitoneal injection or intravenous injection can elicit anti-tumor activity for celastrol *in vivo* [6,7,8]. Not much literature has addressed the low aqueous solubility of celastrol, hence, we intended to explore a oral administration formulation for enhanced oral absorption of celastrol.

Liposomes are small artificial vesicles of spherical shape with a membrane composed of phospholipid bilayers [9]. They can consist of natural nontoxic phospholipids and cholesterol in the form of one or multiple concentric bilayers capable of encapsulating hydrophilic and hydrophobic drugs. Among all the nanomedicine platforms, liposomes have demonstrated one of the most established nanoplatforms, with several FDA-approved formulations for cancer clinical trials with respect to the impact on tumorigenesis to date because of their size, biodegradability, hydrophobic and hydrophilic character, low toxicity and immunogenicity [10]. Several conventional chemotherapeutic agents such as doxorubicin, paclitaxel and cisplatin with low solubility in the aqueous phase have been successfully encapsulated into pegylated liposomes [11] and have been approved for clinical use in a variety of malignant tumors, such as Kaposi’s sarcoma, breast and ovarian cancers [12].

In this study, we established and characterized the optimized formulation of liposomal celastrol. Subsequently, the effect of liposomes on the intestinal absorption of celastrol was investigated by using the four-site rat intestinal perfusion model, which is recognized by the FDA as a viable model of human intestinal absorption. Moreover, the tumor-bearing model was also applied to examine whether celastrol-loaded liposomes could amplify the anti-tumor capacity of celastrol.

## 2. Results and Discussion

### 2.1. Preparation and Characteristic of Liposomal Celastrol

Liposome preparation methods such as the film-supersonic method, antiphase evaporating method, film-dispersion method, ether infusion method freeze-drying method and ethanol-injection method have been developed in recent years, and various water-miscible solvents such as acetone, ethanol and methanol have been selected for the preparation of liposomes [13,14]. Since celastrol was better dissolved in ethanol, coupled with the miscibility of ethanol and water in any proportion and lower toxicity, the ethanol-injection method was applied. The main advantage of the ethanol injection method is the possibility to acquire small liposomes with narrow distribution by simply injecting an ethanolic lipid solution in water [15,16], and some industrial preparations have been obtained this way.

*Surfactant Screening.* Surfactant molecules can be inserted into liposome phospholipid bilayer membrane, and soften the bilayer membrane. Consequently, the lipid plasmid stability was enhanced. As shown in Table 1, Tween-80 was selected as the surfactant for the following experiments.

**Table 1:** Screening of surfactants between Tween-80, Poloxamer 188 (P188), PEG400, Sodium Deoxycholate (SD) (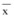 ± s, n = 3).

Surfactants	Tween-80	P188	PEG400	SD
EE%	94.67 ± 3.58	92.36 ± 3.84	76.35 ± 2.03	81.23 ± 2.47

*Influence of Injection Velocity.* The injection rate was varied from 100 to 2,000 µL/min. Lipid concentration and cholesterol percentage were kept constant. According to Table 2, the injection velocity has no significant effect on the encapsulation efficiency and liposome particle sizes. Schubert had also found that the solvent injection technique accounted for lipid precipitation and the rapid diffusion of the solvent across the solvent-lipid interface with the aqueous phase, regardless of the organic solvent injection velocity [17]. Low velocity will accelerate the lipid oxidation. High velocity will result in uneven distribution of particle size. The injection velocity was fixed at 500 µL/min for the following experiments.

**Table 2:** Study of injection velocity of organic phase (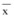 ± s, n = 3).

Injection velocity (mL/min)	0.1	0.5	1.0	2.0
EE%	90.81 ± 2.80	94.67 ± 3.58	94.26 ± 3.78	91.31 ± 2.96
Average Size (nm)	71.31 ± 0.43	63.62 ± 0.44	61.53 ± 0.46	97.41 ± 0.71

*Orthogonal Design*. Phospholipids are major components of liposomes, and have a greater impact on the quality of liposomes. The increase of phospholipid concentration in the aqueous phase will enhance the viscosity of the suspension, as well as the particle size, resulting in reduced liposome stability. However, the decrease of phospholipids concentration will result in too low system drug concentration, thus affecting the whole system and the drug loading. We investigated the correlation between phospholipids and other critical factors by way of single factor experiments and orthogonal design (Table 3). After variance analysis and visual analysis, we drew the conclusion that in the aqueous phase of 40 mL, under the condition of soybean phospholipid 300 mg, accompanied with celastrol 30 mg, cholesterol 80 mg and Tween-80 0.5 mg/mL, the encapsulation efficiency could be optimized. We repeated the best formulation three times to get the EE% as 98.06 ± 0.94%. This optimum dose was selected for the remainder of the work.

**Table 3:** Encapsulation efficiency of different batches of celastrol–loaded liposomes prepared using the ethanol injection method.

Batch	Celastrol	Cholesterol	Tween-80	Aqueous Phase Volume	EE
	Weight	Weight	Concentration		
	(mg)	(mg)	(mg/mL) ^a^	(mL)	(% w/w) ^b^
1	10	40	0	20	79.42 ± 2.45
2	10	80	0.5	30	98.98 ± 2.16
3	10	120	1.0	40	89.77 ± 2.43
4	10	160	2.0	50	80.62 ± 2.37
5	20	40	0.5	40	88.54 ± 2.32
6	20	80	0	50	85.35 ± 2.52
7	20	120	2.0	20	79.89 ± 2.61
8	20	160	1.5	30	85.71 ± 2.59
9	30	40	1.5	50	85.38 ± 2.78
10	30	80	2.0	40	93.55 ± 2.59
11	30	120	0	30	84.18 ± 2.45
12	30	160	1.0	20	87.13 ± 2.53
13	40	40	2.0	20	75.60 ± 2.19
14	40	80	1.5	30	95.57 ± 2.24
15	40	120	1.0	50	89.25 ± 2.53
16	40	160	0	40	80.82 ± 2.62

^a^ Tween-80 concentration with respect to aqueous phase volume used in the formulation; ^b^ Encapsulation efficiencies are expressed as mean values ± standard deviations (n = 3).

**Figure 2:**
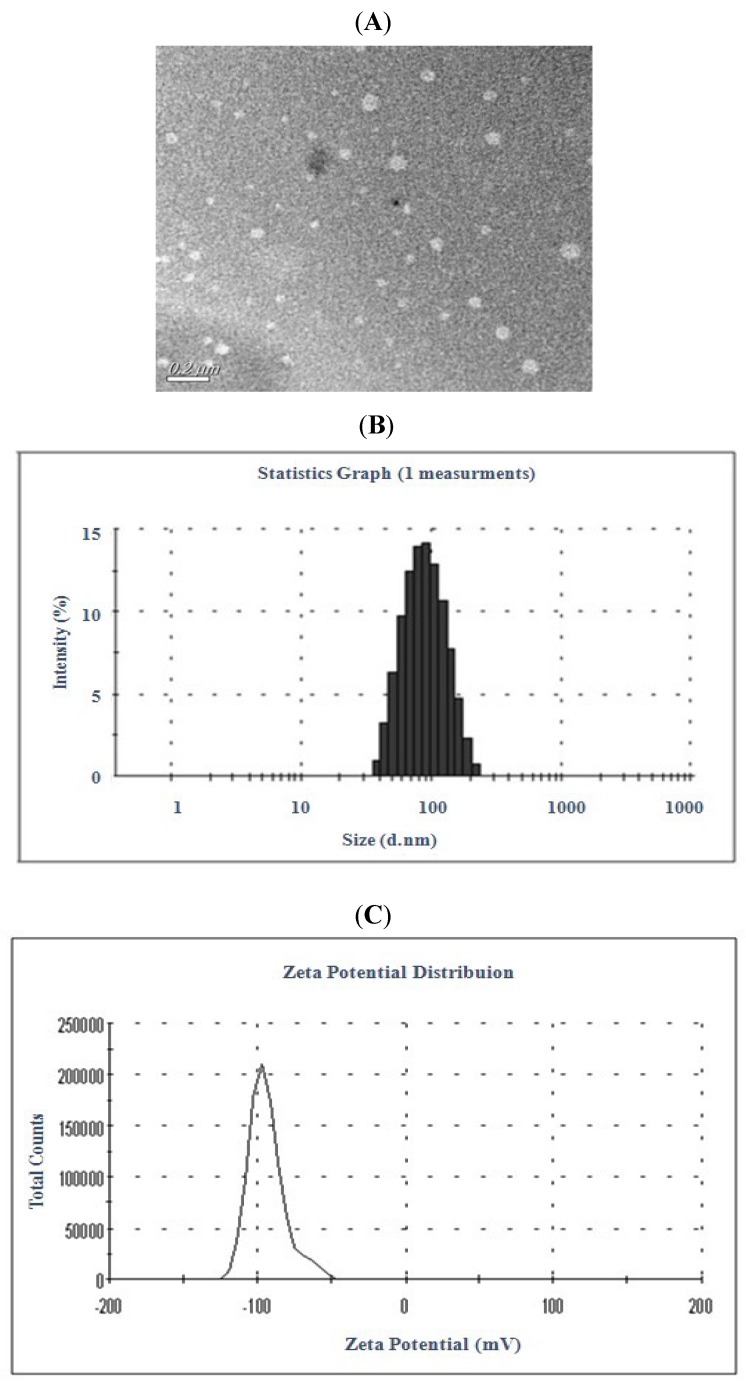
Characteristics of liposomal celastrol. TEM micrographs of celastrol-loaded liposomes prepared by ethanol injection method (**A**); Size distribution and zeta distribution determined by laser particle size analyzer (**B** and **C**).

*Morphology and Zeta Potential Investigation*. The TEM study demonstrated that the particles had almost spherical and uniform shapes and did not stick to each other (Figure 2A). The mean diameter was 89.61 ± 0.53 nm (Figure 2B). Measurement of zeta potential was required to assess the properties of charged particles. In general, nanoparticles could form a stable dispersion when the absolute value of zeta potential was above 30 mV due to the electric repulsion between particles [18]. As shown in Figure 2C the average value of zeta potential of celastrol-loaded liposomes was −(87.7 ± 5.8) mV. This demonstrated that the nanoparticles obtained in this study were a dynamic stable system.

### 2.2. Intestinal Absorption Activity Comparison

Celastrol is one of the most important triterpenoids from Tripterygium, with a variety of physical activities, but no documents had been reported about its intestinal absorption characteristics. By previous study of physical and chemical properties, we found that celastrol was undissolvable in water, with an oil-water partition coefficient Log*P* of 5.63. Therefore, it can be classified as a BCS classification Class IV-type drug, that is, of low solubility and low permeability, suggesting that celastrol may be poorly absorbed by the body. Consequently, the *in-situ* rat intestinal perfusion model test was carried out to further evaluate the intestinal absorption of celastrol when liposomes were used as oral drug delivery systems.

**Figure 3:**
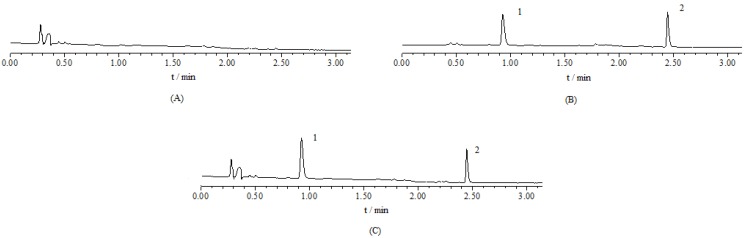
UPLC elution profile of blank control intestinal perfusate (**A**), celastrol (2) and internal standard (**B**), test solution of celastrol (**C**). Testosterone was used as an internal standard (1).

*UPLC Analysis.* The UPLC chromatograms of celastrol are shown in Figure 3. Prednisolone was used as an internal standard. Retention times of celastrol and internal standard were 0.967 min and 2.438 min, and the retention time of the test solution was in consistent with that of standard reference. In addition, endogenous substances did not interfere with the analysis.

*Chemical Degradation Study.* According to the natural log plot of the percentage of drug residues *versus* time, the degradation rate constant k at different pH values was obtained by a first-order kinetic equation. No obvious chemical degradation in 24 h of celastrol was observed from Table 5.

**Table 5:** Investigation for chemical degradation of celastrol.

Concentration (μM)	k (×10^−3^ s^−1^^)^			
	pH 5.5	pH 6.5	pH 7.4	pH 8.0
4	2.11	1.71	1.54	1.39
8.3	2.57	2.49	2.38	2.03
20	3.31	2.17	1.97	1.56

**Figure 4:**
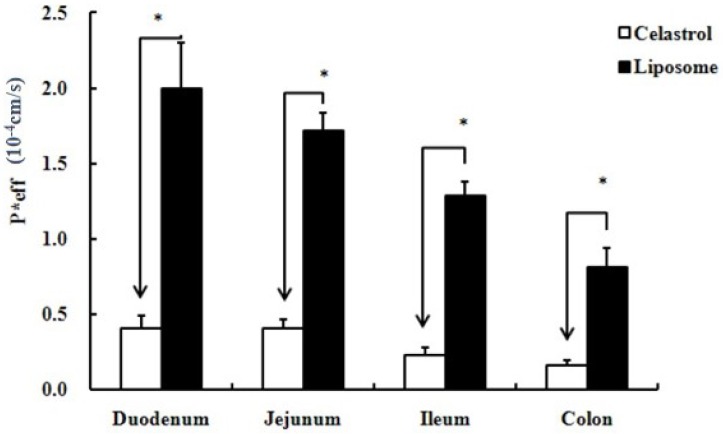
Comparison of P*eff (10^−4^ cm/s) (effective permeability) between four different intestinal segments. Data are expressed as mean ± SD (n = 5). The statistically significant difference is shown by the asterisk symbol, * P < 0.05.

*Comparison of Permeability Efficiency*. Comparing the rat duodenum, jejunum, ileum and colon, the intestinal absorption of celastrol was poor in all four segments, as revealed by P*eff < 0.5 (Figure 4). Pairwise and multiple comparisons were made for the parameters for different segments between liposomal celastrol and the free drug, respectively. Celastrol-loaded liposomes displayed improved absorption capacity in all intestine segments.

### 2.3. Celastrol-Loaded Liposomes Inhibited Tumor Growth

**Figure 5:**
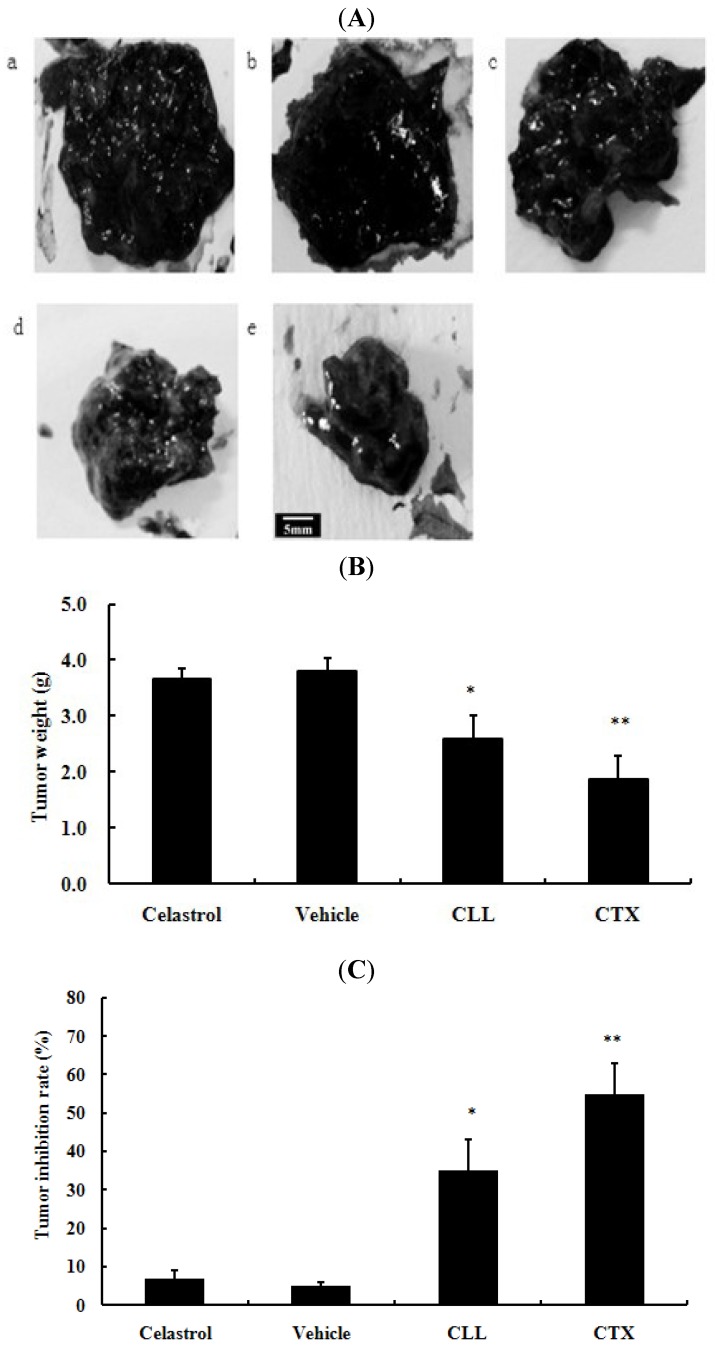
Celastrol-loaded liposomes attenuated tumor growth *in vivo*. (**A**) are presentative figures of tumors excised from C57BL/6 mice. a: blank control group with 0.9% NaCl; b: 2 mg/kg celastrol in 0.2% CMC-Na; c: the vehicle, namely, 2 mg/kg drug-free liposomes; d: 2 mg/kg celastrol-loaded liposomes (CLL), a–d were intragastrically administered every day; e: 20 mg/kg cyclophosphamide (CTX) by intraperitoneal injection. Tumor weight were summarized as shown in (**B**). Tumor inhibition rates accounted for tumor weight were shown in (**C**). Bar = 5 mm. * p < 0.05, ** p < 0.01 versus 0.9% NaCl blank control group.

To assess whether celastrol-loaded liposomes may foster antitumor activity, we have monitored the improvement in the inhibition rate (Figure 5).

In Figure 5A, tumor volumes in the celastrol-loaded liposomes and cyclophosphamide (CTX) groups were visibly smaller than that of the 0.9% NaCl group. As can be seen from Figure 5C, the vehicle (2 mg/kg drug-free liposomes) and original celastrol group exhibited minor effect on tumor growth, while tumor inhibition rates (%) of the positive control (CTX) and celastrol-loaded liposomes groups versus the 0.9% NaCl group were 55.1 ± 8.8, 34.9 ± 7.7. Celastrol-loaded liposomes group and vehicle group did not exhibit much influence on mice weight compared with the 0.9% NaCl group (data not shown).

## 3. Experimental

### 3.1. Chemicals

Celastrol (purity > 98%) was purchased from National Institute for the Control of Pharmaceutical and Biological Products (China). Soybean phospholipid, Tween-80, Hanks’ balanced salt solution (HBSS; powder form) were purchased from Sigma-Aldrich (St. Louis, MO, USA). UPLC grade acetonitrile and acetic acid from Tedia Co. (Fairfield, OH, USA) were used as mobile phase. Sephadex G-50 was obtained from Pharmacia (Uppsala, Sweden). All other materials (typically analytical grade or better) were used as received.

### 3.2. Animals

Male Sprague-Dawley rats (250–300 g) and male C57BL/6 mice (18–20 g) of 6–8 weeks of age were obtained from the SLEK Lab Animal Center of Shanghai (Shanghai, China). They were maintained on a 12 h light/dark cycle at the temperature of 25 ± 2 °C and relative humidity of 50 ± 10% with water *ad libitum*.

### 3.3. Liposome Preparation by Ethanol Injection Method

As celastrol is soluble in ethanol, a modified ethanol-injection is used to prepare celastrol-loaded liposomes. The required amounts of soybean phospholipids and cholesterol were dissolved in ethanol. The resulting organic phase was gently injected to the 55 ± 2 °C aqueous phase under magnetic stirring. Spontaneous liposome formation occurred as soon as ethanolic solution was in contact with the aqueous phase (phosphate buffered saline, pH 7.0). The liposome suspension was then kept under stirring for at room temperature to remove the traces of solvent [19].

### 3.4. Morphological Study by Transmission Electron Microscopy

The liposome suspension was imaged by using a Hitachi H-7650 transmission electron microscope (TEM, Hitachi, Tokyo, Japan). A drop of the liposome suspension was placed onto a carbon-coated copper grid, forming a thin liquid film. The films were negatively stained with 2% phosphotungstic acid solution for 1 minute. The excess of phosphotungstic solution was removed with a filter paper and then the sample was dried in the air before TEM observation [20].

### 3.5. Particle Size and Zeta-Potential Analysis of Liposomes

The average diameter of particles of liposomes was measured by photon correlation spectroscopy (PCS, Zetasizer 3000HSA, Malvern Instruments Ltd., Worcestershire UK). The zeta potential of particles of liposomes was measured by a Malvern Zetasizer Nano ZS90. Each sample was diluted with distilled water until the appropriate concentration of particles was achieved, and each sample was measured in triplicate. All measures were performed in triplicate at 25 °C.

### 3.6. Determination of Encapsulation Efficiency

Encapsulation efficiency was calculated after separation of the non-entrapped drug using the mini-column centrifugation method [21]. This method was able to separate all the free drug as evidenced by the absence of any drug in the centrifugate in the two stages. Liposomes can be recovered from the first or the first and second stages of centrifugation [22,23]. Sephadex G50 was swollen in distilled water for at least 12 h and stored at 4 °C. To prepare the mini-columns, a little cotton was inserted in the bottom of the barrels of 2 cm^3^ injection syringes which were then filled with gel. Excess water was centrifuged off at 1,500 rpm for 3 min, and 1 mL water was added, then centrifugation repeated thrice. Afterwards, drug-free liposome suspensions were added for presaturation, and celastrol-loaded liposomes were added followed by centrifugation as before. The mini-column was eluted by water, and then centrifugation repeated twice. The encapsulation efficiency was calculated as a percentage of the initial drug added. The resulting solution was analyzed by UPLC as described in 5.9. The EE% (encapsulation efficiency) could be calculated by the following equation:

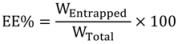

where W_Total_, W_Entrapped_, were the weight of total drug and the weight of entrapped drug.

### 3.7. Animal Surgery

The surgical procedures were approved by the Animal Ethics Committee of Jiangsu Provincial. After overnight fasting, rats were anesthetized. The small intestine was exposed by midline incision; the intestinal lumen was then gently flushed to remove intestinal content and each of the four segments (duodenum, upper jejunum, terminal ileum, and colon) of the intestine was cannulated with two cannulaes. The outlet of each segment was secured by ligation with silk suture. After cannulation, the intestine was carefully arranged and continuously monitored to avoid kinks and ensure a consistent flow. Saline-soaked cotton was used to cover opened body cavities to prevent loss of fluids [24].

### 3.8. Four Site Single-Pass Rat Intestinal Perfusion Experiment

A single-pass intestinal perfusion technique was used. To keep the temperature of the perfusate constant, the inlet cannulae was kept warm by a 37 °C circulating water bath. A flow rate of 0.2 mL/min was used (Harvard Apparatus, Cambridge, MA, USA). The first 30-min presteady-state outlet perfusate was discarded, which represents the stabilization period to reach steady state. Subsequently, samples were collected from the outlet cannulae every 30 min afterward. The outlet concentrations of celastrol in the perfusate were determined by UPLC.

### 3.9. UPLC Analysis of Intestinal Perfusate

Before UPLC analysis, perfusate samples of each segment (400 μL) were mixed with ethanol (100 μL) containing 20 μM prednisolone as an internal standard and were vortexed for approximately 30 s. The mixtures were centrifuged at approximately 14,000 rpm for 15 min, and the supernatants were then analyzed by UPLC. Briefly, the chromatographic system was an Acquity UPLC (Ultra Performance Liquid Chromatography) system (Waters, Milford, MA, USA) with photodiode array detector and Empower software, a Waters UPLC BEH C18, 1.7 μm, 2.1 × 50 mm column, and a linear gradient mobile phase at a flow rate of 0.3 mL/min. Mobile phase consisted of acetonitrile (A) and water containing 1% acetic acid (B). A gradient program was used as follows: 0~0.5 min, 30% A, 0.5~3.2 min, 30%~70% A. The detection wavelength was at 425 nm and the injection volume was 5 μL. The column temperature was set to 35 °C.

### 3.10. Perfusate Preparations and Stability Test of Celastrol in the Perfusate

Celastrol was dissolved with anhydrous ethanol (1 mL) and then 10% Tween 80 solution (5 mL) was added dropwise. After mixing, the solution was diluted with HBSS (pH 7.4) to 20 μM mass concentration of celastrol, namely celastrol perfusate. Celastrol liposome was diluted with HBSS solution to 20 μM mass concentration, which was the celastrol liposomes perfusate. To investigate the stability of celastrol in the perfusate, three samples were incubated in the 37 °C water bath, and samples were measured at 0, 1, 2, 4, 8, 12, 24 h to determine the celastrol content. The first-order kinetic equation was applied to study the degradation reaction constant k, which could reveal the stability of celastrol.

### 3.11. Data Analysis

Briefly, P*_eff_ (effective permeability) is a representation of the intestinal membrane permeability in the perfused rat intestinal model. P*_eff_ of the compounds are calculated as our previous publication describes [25].

### 3.12. Effect of Celastrol-Loaded Liposomes on Tumor Growth in C57BL/6 Mice

The Lewis Lung Carcinoma cells purchased from American Type Culture Collection (Manassas, VA, USA) were maintained in RPMI 1640 supplemented with 10% fetal bovine serum and antibiotics. Lewis cells were trypsinized, resuspended in PBS, and injected into the right anterior limb by the subcutaneous (s.c.) injection of cell suspension (1 × 10^6^). The indicated compounds [2 mg/kg celastrol in 0.2% CMC-Na, 2 mg/kg celastrol-loaded liposomes, 2 mg/kg drug-free liposomes and 0.9% NaCl] were intragastrically administered every day, respectively. Cyclophosphamide (20 mg/kg) was given every day by intraperitoneal injection (i.p.). Body weight and tumor growth were monitored every 2 days. After 16 consecutive days, the tumors were excised and weighed [26].

### 3.13. Statistical Analysis

Data are mean ± standard deviation (S.D.) from three independently performed experiments. The statistical significance was examined using the one-way analysis of variance (ANOVA) followed by Dunnett’s test. P values less than 0.05 was considered statistically significant.

## 4. Conclusions

In conclusion, the celastrol-loaded liposome formulation had good ability to encapsulate drug and elicited favorable physicochemical characteristics. The intestinal absorption and antitumor capacity of celastrol was significantly enhanced by using liposomes. These results suggest that liposomes could be a promising perioral carrier for celastrol.
